# Extra-Renal Wilms' Tumor: A Rare Diagnosis

**Published:** 2015-05-01

**Authors:** Nirali Chirag Thakkar, Yogesh Kumar Sarin

**Affiliations:** Department of Pediatric Surgery Maulana Azad Medical College, and associated Lok Nayak Hospital, New Delhi.

**Keywords:** Wilms' tumor, Extra-renal, Pelvic mass

## Abstract

The diagnosis of extra-renal Wilms’ tumor is often missed at initial clinical presentation leading to a delay in initiating appropriate therapy. A 5-year-old girl presented with a 3-week history of a painless lump in the pelvis. Radiological investigations suggested an ovarian neoplasm. Tumor markers for ovarian malignancy were in normal range. Trucut biopsy also suggested the possibility of an ovarian neoplasm. The tumor was excised and final histopathology revealed it a Wilms’ tumor.

## CASE REPORT

A 5-year-old girl presented with a painless lump in abdomen for 3 weeks. On examination, a 10cm x 8cm, smooth and firm mass occupying the lower abdomen was found. Ultrasound showed a large predominantly solid mass of size 8cm x 7.3cm x 7cm in the pelvis on the right side, separate from the bladder, suspected of ovarian origin. Contrast enhanced CT scan showed the tumor in retroperitoneum with no obvious invasion of surrounding structures. Right kidney was mildly hydronephrotic and separate from the mass (Fig. 1). Levels of α-fetoprotein and HCG were normal. Trucut biopsy showed round to oval cells with eosinophilic cytoplasm with evenly distributed nuclear chromatin, arranged in tubular and acinar pattern with infrequent mitosis and no necrosis, suggesting possibility of ovarian tumor (Fig.2).

**Figure:**
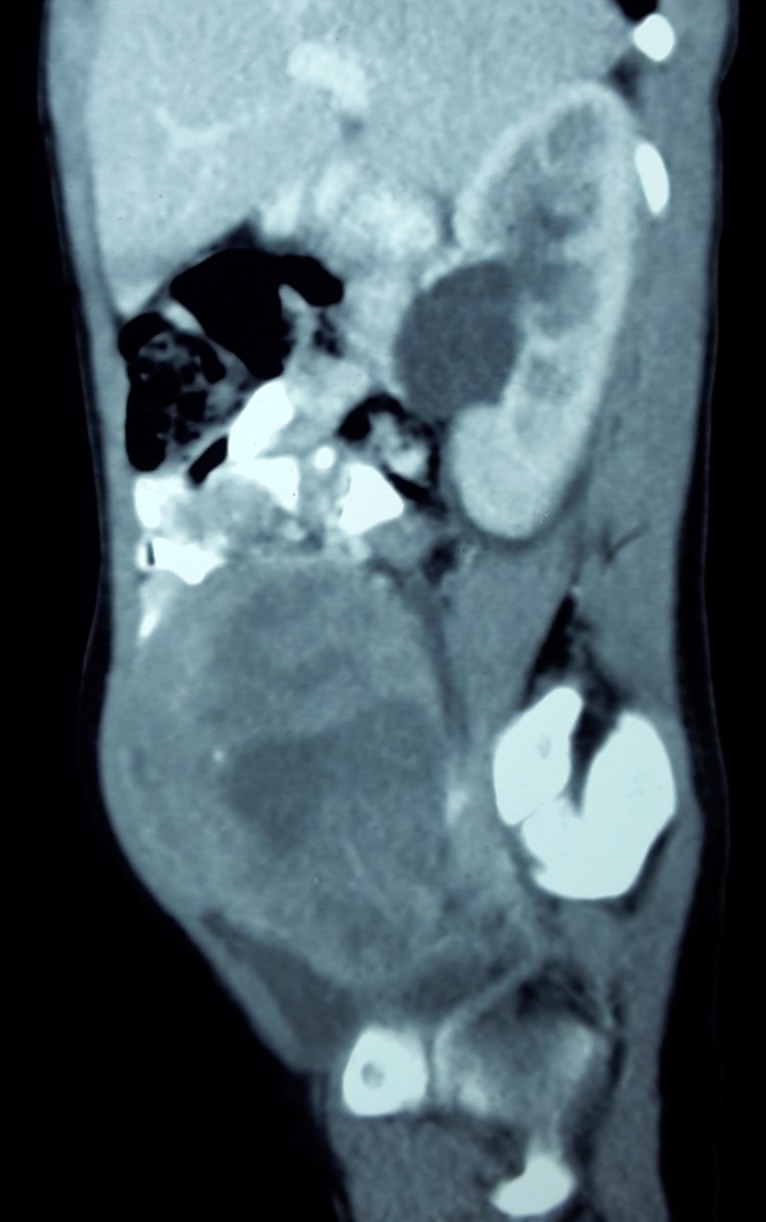
Figure 1: CECT showing a pelvic mass with a hydronephrotic kidney separate from the mass.

**Figure:**
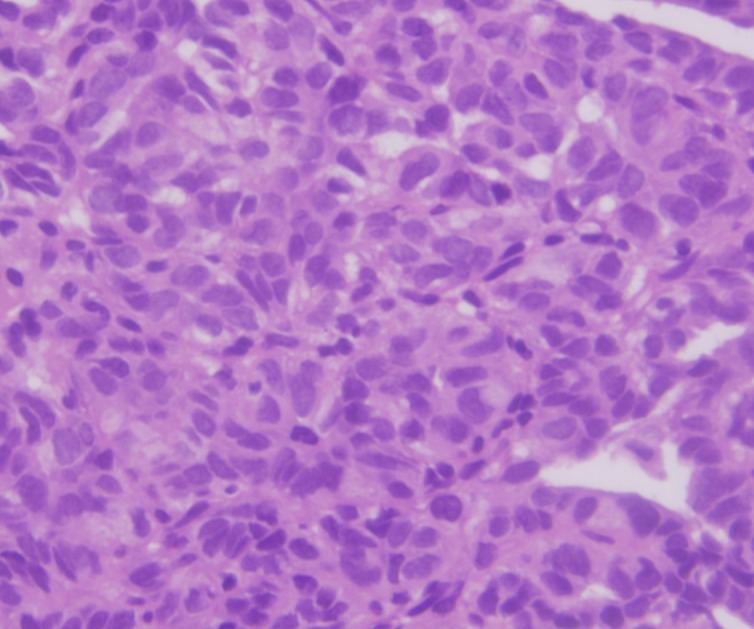
Figure 2: Trucut biopsy showing round to oval cells with eosinophilic cytoplasm and stromal elements.

Intraoperatively, a 10cm x 10cm tumor in the retroperitoneum, adherent to the right ureter and well demarcated from the ovaries, kidneys and other surrounding structures was found (Fig. 3). Only near total excision of the tumor could be done because it was adherent to the ureter. Histopathology was reported as Wilms’ tumor – favorable histology (Fig 4). There was no evidence of vascular invasion. It was covered by a thin fibrous capsule that was focally infiltrated by the tumor. The fibrovascular and adipose tissue surrounding the ureter also showed focal deposits of malignancy. The patient was thus diagnosed to have extra-renal Wilms’ tumor (ERWT), Stage III – favorable histology. She has completed post-operative radiotherapy and chemotherapy as per NWTS protocol. She is now disease free and on regular follow-up.

**Figure:**
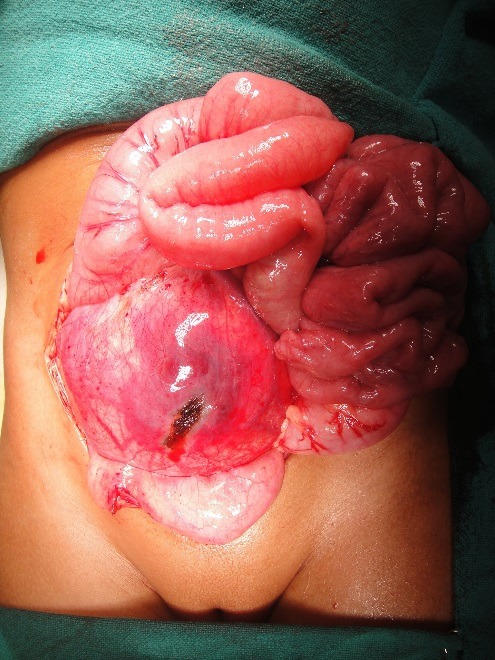
Figure 3: Intraoperative picture.

**Figure:**
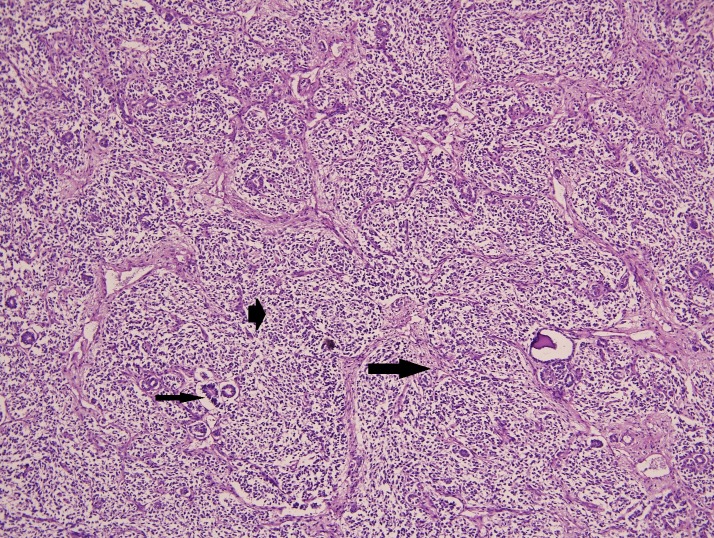
Figure 4: Final histopathology showing epithelial (thin arrow), blastemal (arrow head) and mesenchymal (thick arrow) components characteristic of Wilms’ tumor.

## DISCUSSION

ERWT is a very rare disease, accounting for 0.5-1% of all cases of Wilms’ tumor.[1] The diagnosis is made after a primary renal malignancy has been ruled out. The exact origin of the tumor is not clear. It has been associated with ectopic nephrogenic rests.[2] The various possible tissues of origin include ectopic metanephric blastema [3], primitive mesodermal tissue of mesonephric duct remnants [4], as well as from malignant transformation of cells with persistent embryonal potential (Connheim’s cell-rest theory).[1]

This tumor is more commonly seen in females.[5] It has been reported in all ages ranging from one month to 77 year.[6,7] Extra-renal locations reported include endocervix, uterus, testis, retroperitoneum, skin and thorax. Most cases present with a palpable solid abdominal mass.[8] The prognosis is similar to that of renal WT [6]; staging and treatment is done as per the NWTS protocol.[1]

The differential diagnosis of ERWT is often not considered pre-operatively in such cases. Often there is a significant delay between the initial presentation of the patients and the initiation of appropriate therapy. Most cases are diagnosed postoperatively. Thus clinicians should have a high index of suspicion when dealing with tumors at aberrant locations.

## Footnotes

**Source of Support:** Nil

**Conflict of Interest:** None declared

